# Identification of the Potential Correlation between Tumor Protein 73 and Head and Neck Squamous Cell Carcinoma

**DOI:** 10.1155/2022/6410113

**Published:** 2022-06-15

**Authors:** Yuming Chen

**Affiliations:** Stomatological Hospital, Southern Medical University, Guangzhou 510280, China

## Abstract

**Background:**

Head and neck squamous cell carcinomas (HNSC) are common malignant tumors with a high occurrence and poor prognosis. Tumor protein P73 (TP73) plays an integral role in a wide range of human malignancies, but its gene expression profile, prognostic value, and potential mechanisms in HNSC remain to be comprehensively explored.

**Objective:**

This research aimed to elucidate the potential relationship between TP73 and HNSC through bioinformatics analysis.

**Methods:**

The Cancer Genome Atlas (TCGA) database was queried to investigate the regulatory role of TP73 in HNSC. The survival probabilities linked to TP73 mRNA were determined via the Kaplan-Meier analysis using R packages. Subsequently, the association of TP73 with several clinical subgroups and immunological subtypes was studied using a cohort from the TCGA-HNSC. Functional analyses were used to identify the potential signaling pathways enriched by the correlated genes of TP73. The relationship between TP73 and immunological aspects, including immune cells, immune inhibitor genes, immune stimulator genes, and tumor immune microenvironment, were investigated.

**Results:**

This study showed that the protein and mRNA levels of TP73 in HNSC patients were significantly higher than those in normal tissues. Elevated TP73 expression was related to a better survival outcome in HNSC patients. The TP73 gene was an independent prognostic factor for overall survival in HNSC samples. TP73 was mainly involved in DNA replication, ribosome, apoptosis, mismatch repair, and folate biosynthesis. TP73 was found to be positively correlated with the majority of tumor infiltrating immune cells and immunoinhibitory genes in HNSC.

**Conclusions:**

Integrative bioinformatics and statistical analyses displayed that TP73 might serve as a novel marker for the diagnosis and prognosis of HNSC. TP73 modulates immune cells in the tumor microenvironment of HNSC patients, thereby bearing significance for HNSC immunotherapy.

## 1. Introduction

The p53 gene guard family (including TP53, TP63, and TP73 genes) is involved in tumorigenesis and development by coordinating cell proliferation, death, and differentiation [1]. Unlike frequently mutated TP53, TP73 shows a low mutation rate of 0.6% in primary tumors [2]. Generally, TP73 is considered a tumor suppressor and plays a compensatory role in TP53 mutant tumors. At mild DNA damage stages, TP73 can transcriptionally activate TP53 targets and promote cell cycle arrest, while at irreparable DNA damage stages, it stimulates apoptosis or senescence programs [3].

TP73 exists two functional opposable subtypes, TAp73 and DNp73, imbalance of which is often observed in the process of tumorigenesis [4, 5]. TAp73 behaves as a tumor suppressor and exerts pro-apoptotic effects, while DNp73 lacks N terminus transactivation domain and acts as an oncogene [6]. Therefore, TP73 may suppress or promote tumor growth in different kinds of cancers [7]. According to previous studies, increasing TP73 expression predicted a favorable survival outcome in cervical cancer patients [8], but conversely was associated with an aggressive bladder cancer phenotype [9]. Additionally, aberrant TP73 expression has been linked to hematological malignancies and their poor prognosis [10]. Unregulated TP73 was shown to convert fibroblasts into tumorigenic cells and promote the proliferation of hepatocytes and the progression of early hepatocellular carcinoma [11]. Revealing the molecular mechanisms of TP73 expression in tumors will contribute to understanding TP73 roles, specifically its pro-apoptotic function.

Head and neck squamous cell carcinomas (HNSC) consist of a group of malignancies affecting the mucosal lining in different anatomical regions of the upper aerodigestive tract. HNSC is prone to recurrence and metastasis, so the prognosis is still poor, with only approximately 40%-50% of them surviving within 5 years [12]. The occurrence and development of tumors involve various genetic changes and a variety of physiological changes. Accumulating studies have shown that the TP73 gene is closely implicated in tumor biology. In TP53-mutated HNSC cell lines, the TAP73 tumor suppressor molecule was highly expressed in some cell lines and did not exert its tumor suppressor effect [13]. Immunostaining of oral squamous cell carcinoma (OSCC) revealed that TP73 was a valuable biomarker in diagnosing and monitoring high-risk precancerous lesions in the oral epithelium [14]. However, whether abnormal expression of TP73 occurs in HNSC and its specific molecular mechanisms and translational significance have not yet been elucidated.

The use of bioinformatics in tumorigenesis and development has become an essential mode of oncological research. Our study is the first to use various available databases to bioinformatically analyze the TP73 gene in HNSC patients. The abnormal transcription of TP73 gene in HNSC and its relationship to the clinicopathological parameters among HNSC patients were investigated by leveraging the TCGA database. In addition, survival curve analyses were performed to assess the association between abnormal transcription of TP73 and survival prognosis of HNSC patients. The genes correlated with TP73 were analyzed and functional enrichment analyses were carried out to decipher the activities and pathways that may be affected. Our work shows TP73 could serve as a biomarker of prognosis and displayed possible biological roles in HNSC pathogenesis, thereby making it a highly valuable prospective target for therapy in HNSC.

## 2. Methods

### 2.1. Expression of p53 Family in HNSC Patients

With the help of cBioportal database (https://www.cbioportal.org/), genomic characteristics of tumors can be examined from the DNA level by researchers. After logging into the cBioPortal web, we selected “Head and Neck Squamous Cell Carcinoma (TCGA, Firehose Legacy, Nature 2015 and PanCancer Atlas)” in the “Query” section. “TP53, TP63, TP73” was used as the input to query the variation characteristics. The resulting frequency, mutation types, and copy number changes could be viewed within Oncoprint. TP53, TP63, and TP73 3D structure can be displayed through the “Mutation” module. Plots were selected to analyze the relationship between TP73 copy number changes and gene expression levels in HNSC.

### 2.2. Analysis of Abnormal Levels in Three p53 Family of HNSC

HNSC patients with grade 3 HT-seq data were acquired from TCGA database in FPKM format. Based on TCGA-HNSC data, following analysis of 546 samples, which included 502 HNSC tumors and 44 healthy samples, was performed. Briefly, RNAseq data with FPKM were transformed into log2. The mRNA expression of TP53, TP63, and TP73 in HNSC was analyzed and visualized by using ggplot2 package in R. Samples with no clinical information were excluded. We conducted analysis on both unpaired and paired samples accordingly.

### 2.3. Correlation Coefficient Analysis of p53 Family Genes in HNSC

Pearson's correlation coefficient analysis was adopted to identify the correlation of p53 family genes in HNSC. The visualization of a heat map was based on the *r*- and *P*-value analyzed via ggplot2 package in R.

### 2.4. Abnormal TP73 Expression in Pan-Cancer Containing HNSC

UCSC XENA for RNAseq analysis was processed by Toil method for TCGA as well as GTEx [15]. The TP73 mRNA expression was examined across multiple tumors and visualized by using ggplot2 package in R. The RNAseq data with the format of TPM (transcripts per million reads) were log2 converted and further analyzed by using Mann–Whitney *U* test.

### 2.5. Evaluation of TP73 mRNA Expression in HNSC

ROC analysis was performed to evaluate the diagnostic value of TP73 in distinguishing the clinical status of HNSC (tumor vs. healthy control) [16]. AUC more significant than 0.9 denotes good accuracy. 0.7-0.9 implies a medium level of precision, 0.5-0.7 indicates a poor level of accuracy, and 0.5 suggests an unintentional outcome.

### 2.6. Correlation between TP73 Expression and Methylation in HNSC

Pearson's correlation coefficient was used to investigate the correlation between TP73 and TCGA-HNSC methylation levels. The analyses were based on the beta methylation 450 data and contained 520 RNAseq data of HNSC samples. 49 methylation sites in total were evaluated, three of which contained too many missing values to match.

### 2.7. TP73 Protein Expression Levels in Cancers and Normal Tissues

We typed TP73 and then selected HEAD AND NECK CANCER in Pathology with the help of Human protein atlas (https://www.proteinatlas.org/). Nasopharynx, thyroid, and tonsils were selected as the representative tissue types of HNSC, and the same antibody (CAB002514) were selected. The TP73 protein levels were also analyzed in other types of cancers and various normal tissues.

### 2.8. The expression of TP73 in HNSC subgroups divided by clinicopathological features

The mRNA expression level of TP73 in these two groups was examined by using a t-test and visualized by box plots. HNSC tumor samples were divided into 2 groups according to the different clinicopathological variables.

### 2.9. Investigation of TP73 Prognostic Value by Survival Analysis

Survival heatmaps were visualized via GEPIA2. The TCGA-HNSC database was used to gather TP73 mRNA expression, clinicopathological data, and general information. The chi-square, Fisher's exact test, and Wilcoxon's rank-sum were applied. The HNSC patients were divided based on the median level of TP73 expression. Categorical variable level frequencies were then calculated for the low and high TP73 gene expression groups.

Survival data on tumor samples were included in the TCGA-HNSC dataset. Kaplan-Meier (KM) curves were used to compare survival for different TP73 patterns via log-rank test. The statistical analysis was performed based on the survival package in R, and KM plots were visualized by using the survminer package in R. Based on TP73 expression levels, tumor data were classified as low- and high-expressing TP73. Specifically, progress-free interval (PFI), overall survival (OS), and disease-specific survival (DSS) events were evaluated.

### 2.10. Subgroup Survival Analysis

This part determined whether overexpression of TP73 mRNA had a significant effect on the OS outcome of HNSC cases by survminer package (version 0.4.9). The statistical analyses were performed via Cox regression. Statistical analyses were conducted with R to obtain KM plots.

### 2.11. Relationship between TP73 Expression and Clinical Characteristics

The HNSC samples were divided into TP73 low- and high-expression groups. The relationship between TP73 expression and 16 clinicopathological features was analyzed by using the Chisq test, Fisher's test, and Wilcoxon's rank-sum test.  Table 1 shows the distribution of TP73 low- and high-expression samples in different subgroups of HNSC patients.

### 2.12. Univariate and Multivariate Cox Regression for Survival Analysis

An analysis of Cox regression was performed to determine a correlation between clinical variables and prognosis. The results were achieved by applying the coxph function in R.

### 2.13. Forest, Nomogram, and Calibration Plots

We constructed forest plots in R using the ggplot2 package so that hazard ratio (HR), 95% CI, and *P* value were obtained. When comparing two instances of a binary feature, the HR is used to determine the relative risk of death. HR>1 implies higher, whereas HR<1 suggests lower.

Combining TP73 expression values with clinical variables to construct a predictive nomogram for HNSC has not been reported. Using the TCGA-HNSC dataset, we created a predictive plotting by means of combining characteristics and gene expression. We used the rms and survival package to draw nomogram survival plots in R. The prognostic type chosen was OS. TNM stages, histological stage, smoking, alcohol history, radiation, primary outcome neck dissection, lymphovascular invasion, TP73 expression, and other characteristics were studied.

Each of Calibration diagram was carried out via rms and survival packages. Fitting the actual observed fractional probabilities at three-time points was plotted to evaluate the model's prediction accuracy for the actual prognostic outcomes. The model has achieved perfect predictions if the 1-, 3-, and 5-year solid lines match the 45° ideal diagonals.

### 2.14. Construction of Protein-Protein Interaction (PPI) and Gene-Gene Interaction (GGI) Network

A PPI network comprising TP73 coexpressed genes was created based on the STRING database (https://string-db.org/, version 11.5). The following are examples of advanced settings: high confidence (0.700), no more than 20 interactors. Analyses of PPI network's TSV file were performed by using NetworkAnalyzer tool in Cytoscape software. Central genes were identified as those with a degree of more than 10 via CytoHubba plug-in. Based on TCGA-HNSC data, the expression pattern and OS outcomes of top 10 hub genes in HNSC were evaluated with ggplot2 R language package.

A TP73-based GGI network was built using the GeneMANIA webserver (http://genemania.org). The TP73 gene was used as input and the principal functions in the network with the fewest FDR values were chosen. TP73 and its 20 associated genes make up the network. A GGI network was constructed, and then the network photos and reports were saved.

### 2.15. Genes Significantly Correlated with TP73 in HNSC

We used the stat package in R in order to perform the correlation analysis regarding TP73. Pearson's correlation analysis was performed to obtain the significantly correlated genes of TP73 in HNSC. The protein-coding genes were selected from the correlated genes [17].

### 2.16. The Top 10 Associated Genes in TP73 Plotted in a Heat Map

Following the TP73 correlated genes determination, a list of top 10 genes sorted by cor-Pearson value in descending order was acquired, which were considered the top 10 positive genes. And a list of top 10 negative genes was sorted by cor-Pearson value in ascending order. The expression patterns of these total 20 related genes in HNSC samples were drawn by using a heatmap.

### 2.17. Functional Enrichment Analysis

A clusterProfiler package (version 3.14.3) was performed to identify the function enriched by significantly correlated genes of TP73. The adjusted *P* values were calculated by Benjamini and Hochberg statistical method. By setting a threshold of *P* adj<0.05 and *q*-value<0.20, GO terms (e.g., BP (biological process), CC (cellular component), and MF (molecular function)) and KEGG pathways strongly enriched by the top 200 positive and top 200 negative TP73-correlated genes were identified. Bubble plots were generated only for the top 30 terms with *P* adjusted values if this threshold setting significantly contained more than 30 terms. Otherwise, all words were utilized for bubble mapping. Bubble plots were created via ggplot2 package in R.

### 2.18. Gene Set Enrichment Analysis (GSEA)

Log2FC (fold change) values of TP73 genes were obtained for GSEA. The differentiated expressed genes (DEGs) dysregulated between HNSC and healthy control samples were identified by using DESeq2 package (version 1.26.0) in R. [18]. GSEA analysis was carried out by using the clusterProfile package in R [19, 20]. Access was obtained from KEGG, WikiPathways (WP), and Reactome (REAC) databases. Significant terms included *P* < 0.05, *q*-value<0.25, and |NES| condition > 1. 30 functions, including all of the 10 functions with negative NES value, and top 20 functions with the highest positive NES value, were shown in Table 2 and visualized by mountain plots.

### 2.19. TP73 Expression and Tumor Immune Infiltrating Cells (TIICs) in HNSC

Pearson's statistical approach investigated the relationship between TP73 and 24 TIICs in HNSC. The GSVA package (version 1.34.0) in the R was used to conduct the analysis [21]. Prof. Gabriela Bindea's work provided the genetic markers for the 24 TIICs [22]. The lollipop plot showed the relationship regarding TP73 expression as well as 24 TIICs of HNSC tissues. For the TIICs with statistically significant correlation with TP73 in HNSC, scatter plots regarding these TIICs were displayed.

### 2.20. The Correlation between TP73 and Surface Markers of TIICs in HNSC

We explored the associations between TP73 and immune marker sets of 16 TIICs based on the TCGA-HNSC database [23]. Accordingly, the cor-Pearson and *P* value were analyzed via ggplot2 in R.

### 2.21. TP73 and Immunomodulator genes in HNSC

23 immunoinhibitor genes and 42 immunostimulator genes were chosen after thorough research of immunomodulator genes in HNSC [24]. Pearson's correlation coefficient was utilized to examine the correlation between TP73 expression and immumodulator genes. TP73's association with each of the statistically significant immunomodulator genes was illustrated using scatter plots. The correlation between TP73 and Estimate-Stromal-Immune score was estimated by the estimate package (version 1.0.13).

## 3. Results

### 3.1. Current Research Flowchart

**Figure 1:**
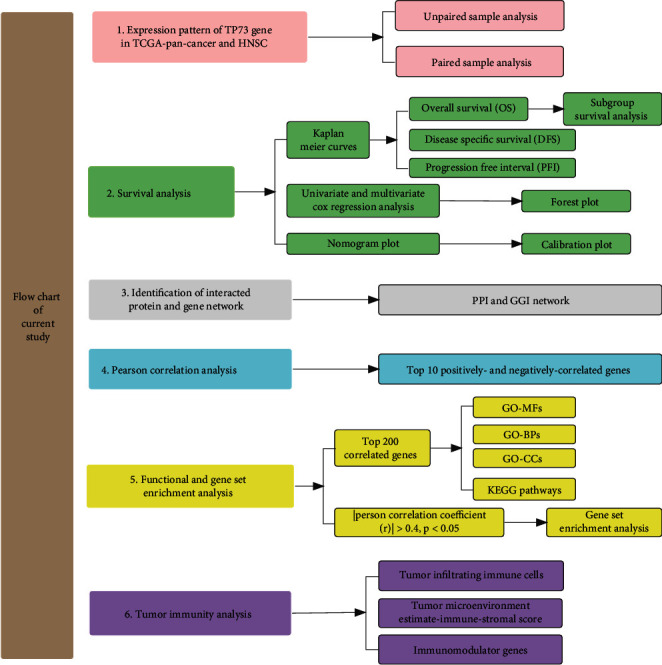
The schematic diagram of the study design.

Figure 1 depicts a flowchart for this study's design visualization. Firstly, an investigation of the TP73 gene in TCGA-pan-cancers and HNSC was conducted. Secondly, to assess the TP73 function in pan-cancer and HNSC, survival analyses were conducted, including KM analysis, univariate, multivariate Cox regression analysis, as well as nomogram plots. Thirdly, the PPI and GGI networks were built to identify the interacted protein and genes. In step 4, Pearson's analysis was used to determine the top ten positively and negatively associated genes. In step 5, we conducted functional enrichment analysis as well as GSEA for investigating the relevant functions of correlated genes. In step 6, we explored the role TP73 played in tumor immunity by investigating TIICs, the tumor immune microenvironment and immunomodulatory genes.

### 3.2. The Mutation Status and Expression of p53 Family Genes in TCGA-HNSC

**Figure 2:**
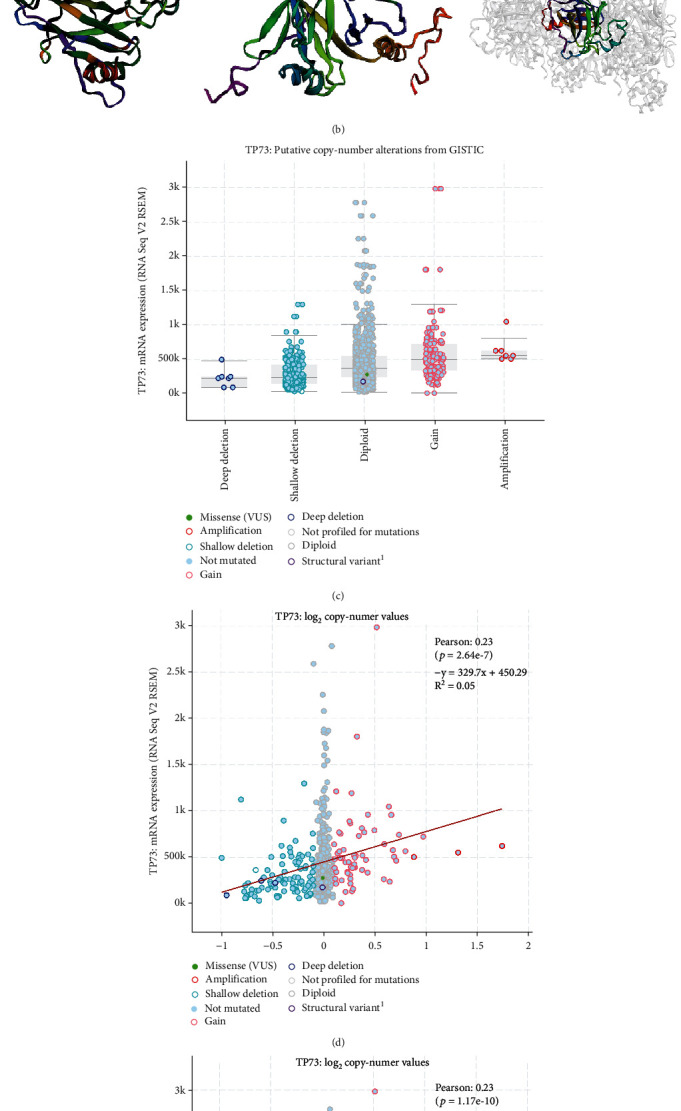
The mutation status of p53 family in TCGA-HNSC. (a) The OncoPrint plotting depicts the prevalence of p53 family mutations within TCGA-HNSCs. (b) 3D structure of the p53 family. (c) Plots showed the putative copy number alterations of TP73 from GISTIC. (d) Plots showed the capped relative linear copy number values of TP73. (e) Plots showed the log2 copy number values of TP73.

The mutation frequency of the p53 gene family in TCGA-HNSC was depicted in an OncoPrint plot (Figure 2(a)). Alteration of the TP53 gene was observable in 70% of HNSC tumor samples (931/1330), whereas alteration of TP63 in 21% and TP73 in 1.3% (276/1330 and 17/1330). According to the mutation type and site, truncating mutation of TP53 and amplification of TP63 and TP73 genes can also be observed.  Figure 2(b) shows the 3D structure of the p53 family genes.  Figure 2(c) shows that HNSC samples harboring TP73 deletion (e.g., deep deletion, shadow deletion) exhibited lower mRNA expression, compared with the HNSC samples that exhibit diploid TP73. There was a significant correlation between TP73 copy number value, and mRNA expression in HNSC samples (Figure 2(d): Log2 copy number values: *r* =0.23, *P* =2.64e^−7^; Figure 2(e): capped relative linear copy number values: *r* =0.23, *P* =1.17e^−10^). This result indicated that mutations and copy number mutations were not the main causes of changes in TP73 mRNA expression among HNSC patients.

### 3.3. Correlation Coefficient Analysis of p53 Family Genes in HNSC

**Figure 3:**
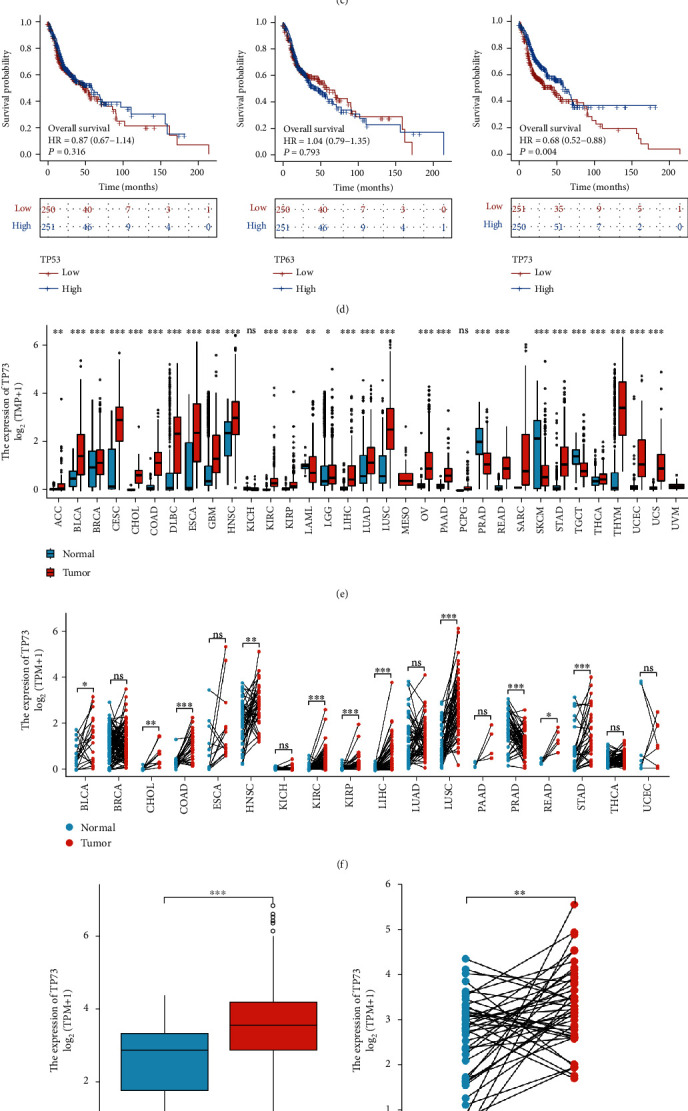
The expression of TP73 in HNSC and pan-cancer. (a) p53 family expression in HNSC and normal tissues was determined via unpaired sample analysis, ∗∗*P* < 0.01, ∗∗∗*P* < 0.001. (b) p53 family expression in HNSC and normal tissues was determined via paired sample analysis. (c) A heat map visualized the correlation coefficient analysis of p53 family genes in HNSC. (d) KM analysis was carried out to investigate the correlation between OS and TP73 gene in HNSC based on TCGA data. (e) TP73 expression in pan-cancer via unpaired sample analysis. (f) TP73 expression in pan-cancer via paired sample analysis. ∗*P* < 0.05, ∗∗*P* < 0.01, ∗∗∗*P* < 0.001. (g) TP73 expression in HNSC via unpaired sample analysis. (h) TP73 expression in HNSC via paired sample analysis. (i) A ROC curve evaluated the diagnostic values of TP73 in predicting clinical status (tumor vs. normal).

Results of Figures 3(a) and 3(b) were consistent, showing that the difference in TP53 expression in HNSC and normal tissues was not statistically significant. However, TP63 and TP73 expression levels in HNSC were upregulated than that in normal tissues based on the TCGA data.  Figure 3(c) presents that a most highly positive correlation was found between TP73 and TP53 in HNSC with an *r* value of 0.414 (*P* <0.001). TP63 and TP73 were also significantly correlated (*r* =0.199, *P* <0.001), while no significant correlation was found between the expression levels of TP53 and TP63 in HNSC (*P* =0.084). Among these three p53 family genes, elevated TP73 expression was strongly correlated with higher OS regarding the HNSC cohort, while TP53 and TP63 genes were not statistically associated with survival outcomes according to the KM survival curves.  Figure 3(d) vividly presents that only the expression difference of TP73 gene has a significant survival probability in HNSC, so this research was mainly focused on it.

### 3.4. Different TP73 Expression in Pan-Cancer Containing HNSC

TP73 mRNA was assessed by using TCGA RNAseq data (Figures 3(e) and 3(f)). Apart from HNSC, TP73 was also found to be remarkably higher in numerous pan-cancers, for example, adrenocortical carcinoma (ACC), breast invasive carcinoma (BRCA), esophageal carcinoma (ESCA), brain lower grade glioma (LGG), liver hepatocellular carcinoma (LIHC), lung adenocarcinoma (LUAD), lung squamous cell carcinoma (LUSC), ovarian serous cystadenocarcinoma (OV), prostate adenocarcinoma (PRAD), rectum adenocarcinoma (READ), stomach adenocarcinoma (STAD), thyroid carcinoma (THCA), thymoma (THYM), and uterine corpus endometrial carcinoma (UCEC). However, TP73 mRNA was statistically decreased in a number of malignancies, including pancreatic adenocarcinoma (PAAD), skin cutaneous melanoma (SKCM), and testicular germ cell tumors (TGCT). Unlike the unpaired sample analyses, the paired sample data showed TP73 has no aberrant differences for BRCA, ESCA, LUAD, PAAD, THCA, and UCEC.

Figures 3(g) and 3(h) prove again the significantly increased TP73 gene expression in HNSC tissues (*P* < 0.001 in unpaired sample analysis and *P* < 0.01 in paired sample analysis). In addition, the ROC analysis was used to assess the diagnostic value of TP73 in HNSC for its diagnostic potential. TP73 could differentiate HNSC samples from normal tissues with moderate accuracy (AUC =0.737, Figure 3(i)).

### 3.5. DNA Methylation Analysis of TP73 in HNSCC

The relationship between TP73 and methylation of HNSC patients is summarized in Table 3. TP73 was positively correlated with 10 methylation sites such as cg22614891, cg18021902, cg19135761, cg26128092, and cg07174627. Additionally, TP73 was negatively correlated with 14 methylation sites such as cg11504517, cg05924583, cg16741710, cg24073122, and cg20611911. The other 22 methylation sites had no significant correlation with the expression level of TP73.

### 3.6. TP73 Protein Expression Levels in Cancers and Normal Tissues

**Figure 4:**
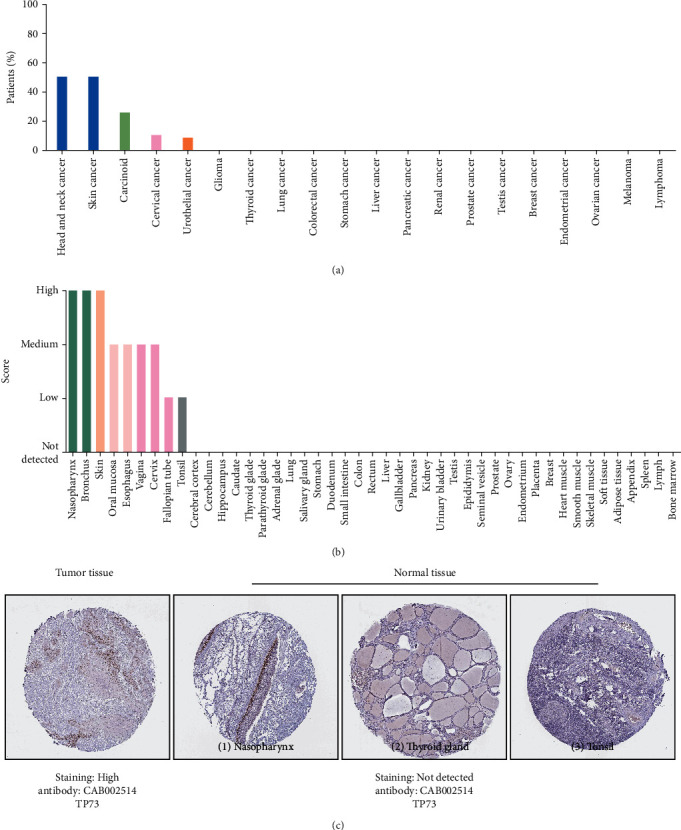
TP73 protein expression levels in cancers and normal tissues. (a) TP73 protein expression levels in various cancers. (b) TP73 protein expression levels in various normal tissues. (c) Results of immunohistochemical showed the expression pattern of TP73 in normal tissues (nasopharynx, thyroid gland, and tonsil) and HNSC.

HNSC along with several skin cancers showed moderate to strong TP73 nuclear positivity. Several cervical and a few urothelial cancers exhibited weak to moderate immunoreactivity, while other cancer tissues were negative (Figure 4(a)). Noticeably, 50% HNSC patients showed high/medium TP73 expression.  Figure 4(b) displays the protein expression of TP73 in various types of healthy tissues. It showed high expression in tonsil squamous epithelial cells and medium in oral mucosa squamous epithelial cells. In consistent with the elevated mRNA expression (Figures 3(g) and 3(h)), TP73 protein was also highly expressed in HNSC but not detected in normal tissues such as nasopharyngeal thyroid and tonsil (Figure 4(c)).

### 3.7. Relationship between TP73 Expression and Clinical Features

**Figure 5:**
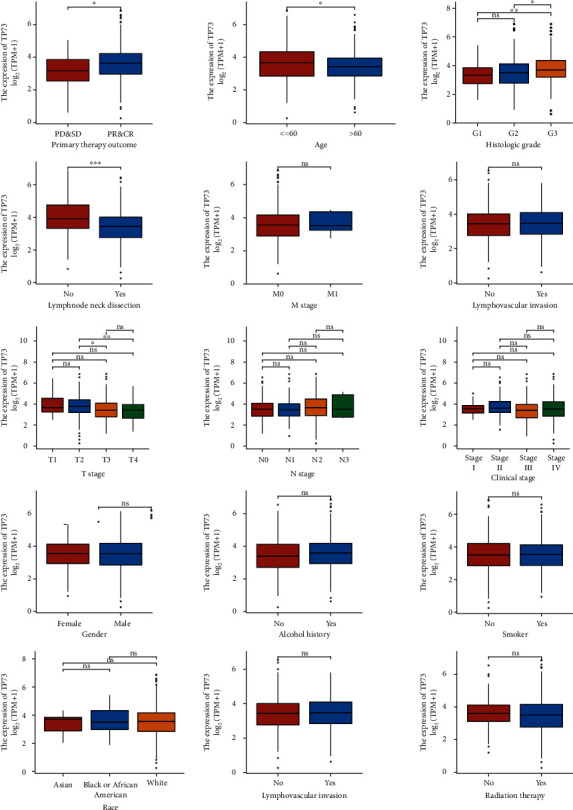
Relationship between TP73 expression and clinical features.

Five clinical variables were significantly associated with the expression level of TP73 gene (Figure 5). The expression of TP73 was significantly higher in the subgroup tumor patients with response to treatment (PR & CR) compared with the subgroup tumor patients without response to treatment (PD & SD, *P* = 0.011). The TP73 mRNA expression levels of HNSC samples with higher histologic grade (G3) were higher than that of samples with lower histological grade (G1: *P* = 0.008, G2: *P* = 0.018). The elevated expression of TP73 was observed in the subgroup tumor patients with younger age (≤60) compared with older age (>60, *P* = 0.035). In addition, TP73 mRNA expression levels of HNSC patients without lymphnode neck dissection were higher than that of patients with lymphnode neck dissection (*P* < 0.001). The TP73 mRNA expression levels of HNSC samples with a T2 stage were elevated than that of samples with a higher T stage (T3: *P* =0.048, T4: *P* = 0.002). However, there was no significant relationship between the other 11 clinical variables (i.e., M stage and lymphovascular invasion) and TP73 gene expression.

### 3.8. Clinical Characteristics of the TCGA-HNSC Patients

According to Table 1, TP73 expression was significantly correlated with histological grade, lymphnode neck dissection, and DSS and PFI events (*P* <0.05), while no significant relationships were observed in other 14 variables.

### 3.9. Survival Analyses of TP73 in Pan-Cancer and Particularly HNSC

**Figure 6:**
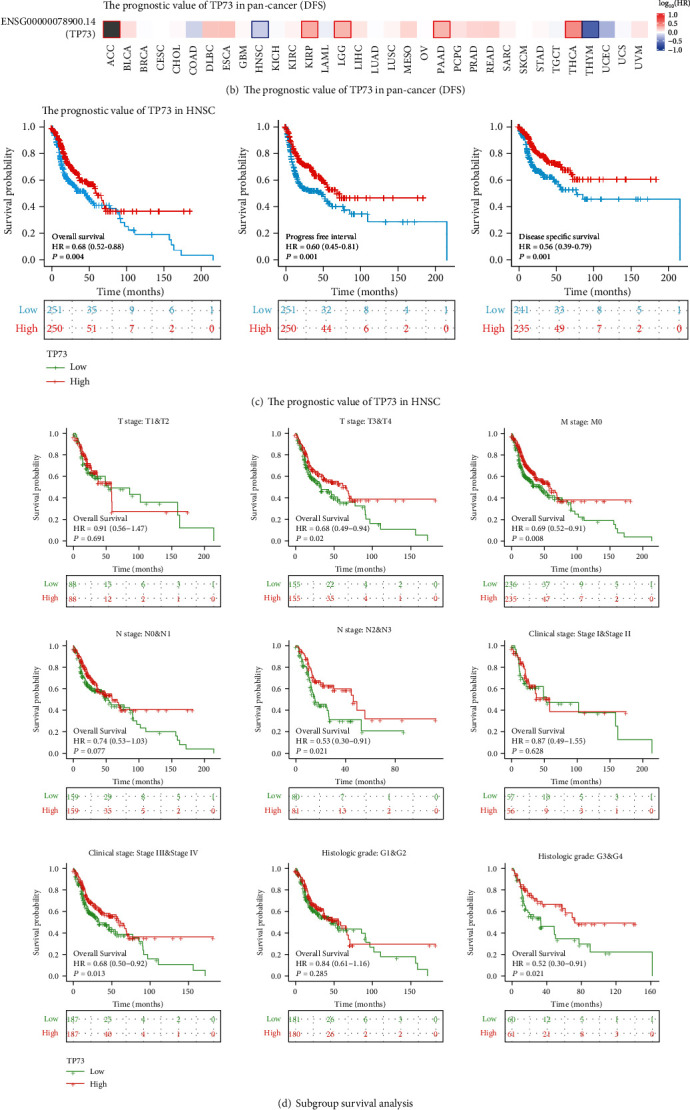
The prognostic value of TP73 in HNSC. (a–b) Heat maps displayed the prognostic impact of the TP73 mRNA on OS and DFS for multiple cancers. The boxes indicate unfavorable (red) as well as favorable outcomes (blue). (c) The prognostic value of TP73 in HNSC regarding 3 prognostic parameters (OS, PFI, and DSS). (d) The association between elevated TP73 mRNA and OS outcome of HNSC using the subgroup survival analysis according to the clinical characteristics.

Figures 6(a) and 6(b) present the survival heatmaps, showing the prognostic impact of TP73 in various cancers. In ACC, KIRC, and LGG, elevated TP73 was associated with a worse OS, while in ACC, KIRC, LGG, PAAD, and THCA, elevated TP73 was associated with a worse DFS. Noticeably, the upregulated TP73 was associated with a better OS and DFS survival outcomes in HNSC patients.

Figure 6(c) indicates the prognostic values of TP73 gene for HNSC patients. There was a significant difference in survival time distribution of three prognostic types (OS, PFI, and DSS) according to different expression groups of TP73, which suggested the overexpressed TP73 may predict a better OS outcome.

### 3.10. Subgroup Survival Analyses

Figure 6(d) suggests that high expression of TP73 did not mean better survival for patients with lower T stages (T1 and T2), while for patients with higher stages (T3 and T4), high expression of TP73 represented greater survival probability. Since there were insufficient samples in M1 subgroup, M stage was not used for performing the subgroup survival analyses. The upregulation of TP73 had a significant impact on OS outcomes in HNSC cases with a higher N stage (N2 and N3, *P* = 0.007), clinical stage (Stage III and Stage IV, *P* = 0.013), and histologic grade (G3 and G4, *P* = 0.021). However, upregulation of TP73 did not exert a statistically significant OS outcome in HNSC cases with a lower N stage (N0 and N1, *P* = 0.077), clinical stage (Stage I and Stage II, *P* = 0.628), and histologic grade (G1 and G2, *P* = 0.285), respectively.

### 3.11. Visualization of Univariate and Multivariate Cox Regressions via Forest Plotting

**Figure 7:**
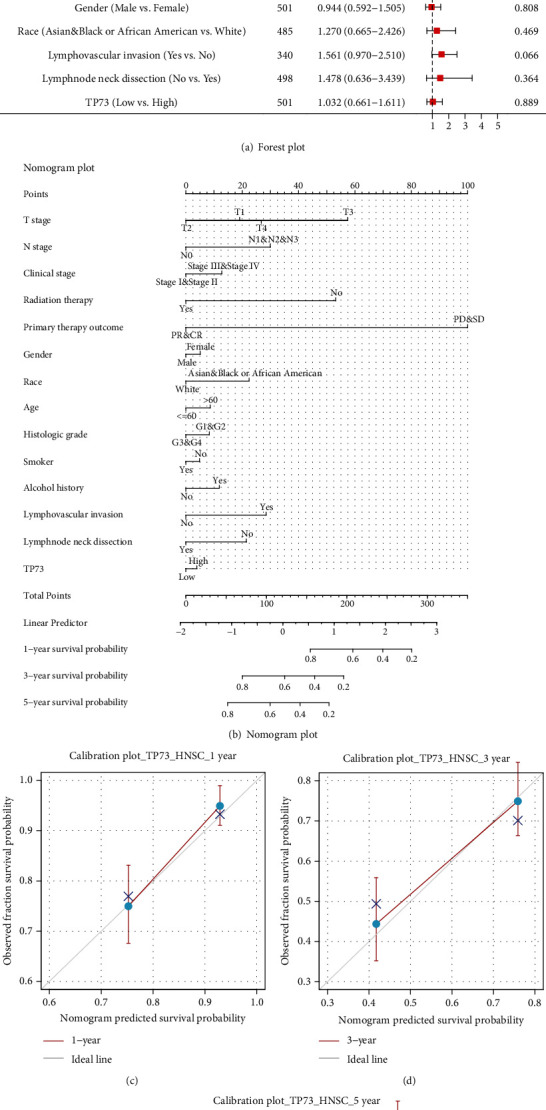
Forest plot, nomogram plot, and calibration plot. (a) Forest plots visualized the Cox regression analysis of TP73 and clinical characteristics. (b) Nomograms predict in HNSC patients at 1, 3, and 5 years. (c-e) Calibration curves for predicting the OS outcome.

In Figure 7(a), univariate analyses results showed that various characteristics are risk factors for death outcome in HNSC patients, including M1 stage (*P* = 0.002), without receiving radiation therapy (*P* = 0.002), primary therapy outcome of PD & SD (*P* < 0.001), and with lymphovascular invasion (*P* = 0.002). Noticeably, an HR of 1.480 for low TP73 expression HNSC patients indicated that the group had a higher risk of death than those high TP73 gene expression (*P* = 0.004).

According to multivariate analyses results, a number of characteristics, for example, high N stage (N1 and N2 and N3) (*P* = 0.029), not receiving radiation therapy (*P* = 0.006), and primary therapy outcome of PD and SD (*P* < 0.001), influenced OS outcomes in HNSC patients. However, the level of TP73 expression did not make a difference in survival outcomes.

### 3.12. Conduction of a Prognostic Model for Risk Estimation

Figure 7(b) predicts the survival prediction for HNSC samples, based on the TP73 gene as well as clinical factors. The deviation correction line in the 1- and 3-year OS calibration plotting is close to the ideal curve, indicating that the prediction results agree well with the observation results (Figures 7(c) and 7(d)). Noticeably, the predicted result did not agree well with actual survival results according to the calibration curve for predicting 5-year OS (Figure 7(e)).

### 3.13. PPI Network Plotting

**Figure 8:**
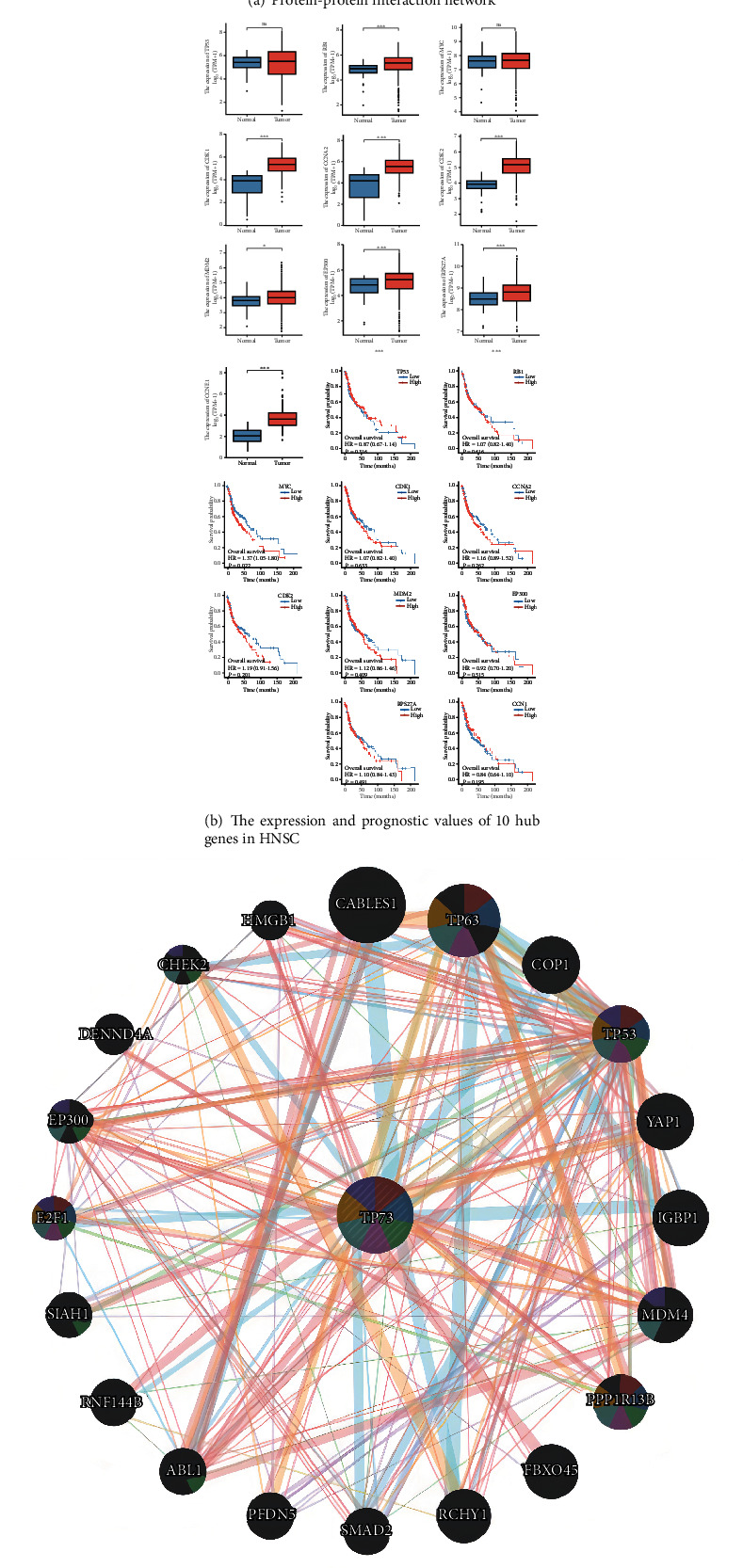
The correlated proteins and genes of TP73. (a) TP73 coexpressed genes constitute a PPI network based on STRING software. (b) The correlations between the top 10 hub genes with TP73 in HNSC patients. (c) GeneMania webserver was used to design the GGI network.

A PPI network of TP73 expressions with 21 nodes and 88 edges was generated, and it has an interaction score >0.7 according to the STRING online database (Figure 8(a)). Apart from TP53, TP73 was also found to interact with other proteins, for instance, MDM2 (MDM2 Proto-Oncogene), YAP1 (Yes1 Associated Transcriptional Regulator), and EP300 (E1A Binding Protein P300).  Figure 8(b) displays the expression and prognostic values of top 10 hub genes in HNSC. Among them, 8 hub genes were significantly upregulated in HNSC, including RB1, CDK1, CCNA2, CDK2, MDM2, EP300, RPS27A, and CCNE1. Regarding prognostic values, only the MYC expression level was significantly related to OS outcomes in HNSC (*P* = 0.022). Elevated MYC expression presented a worse survival probability for HNSC patients.

### 3.14. GGI Network Plotting

According to Figure 8(c), GGI network contained TP73 gene and its 20 interacted genes. These genes contained CABLES1 (Cdk5 and Abl enzyme substrate 1), TP63 (tumor protein p63), COP1 (COP1 E3 ubiquitin ligase), TP53 (tumor protein p53), YAP1, IGBP1 (immunoglobulin binding protein 1), MDM4 (MDM4 regulator of p53), PPP1R13B (protein phosphatase one regulatory subunit 13B), FBXO45 (F-box protein 45), RCHY1 (ring finger and CHY zinc finger domain containing 1), SMAD2 (SMAD family member 2), PFDN5 (prefoldin subunit 5), ABL1 (ABL proto-oncogene 1, nonreceptor tyrosine kinase), RNF144B (ring finger protein 144B), SIAH1 (siah E3 ubiquitin protein ligase 1), E2F1 (E2F transcription factor 1), EP300 (E1A binding protein p300), DENND4A (DENN domain containing 4A), CHEK2 (checkpoint kinase 2), and HMGB1 (high mobility group box 1). The functional enrichment analysis showed that these 20 interacted genes were significantly enriched in several apoptotic signaling pathways, such as regulation of protein insertion into mitochondrial membrane, protein insertion into mitochondrial membrane, positive regulation of mitochondrial outer membrane permeabilization, intrinsic apoptotic signaling pathway, regulation of mitochondrial outer membrane permeabilization, signal transduction by p53 class mediator, and regulation of cell cycle arrest.

### 3.15. Heat Map to Visualize the Top Correlated Genes Expression Pattern of TP73 in HNSC

**Figure 9:**
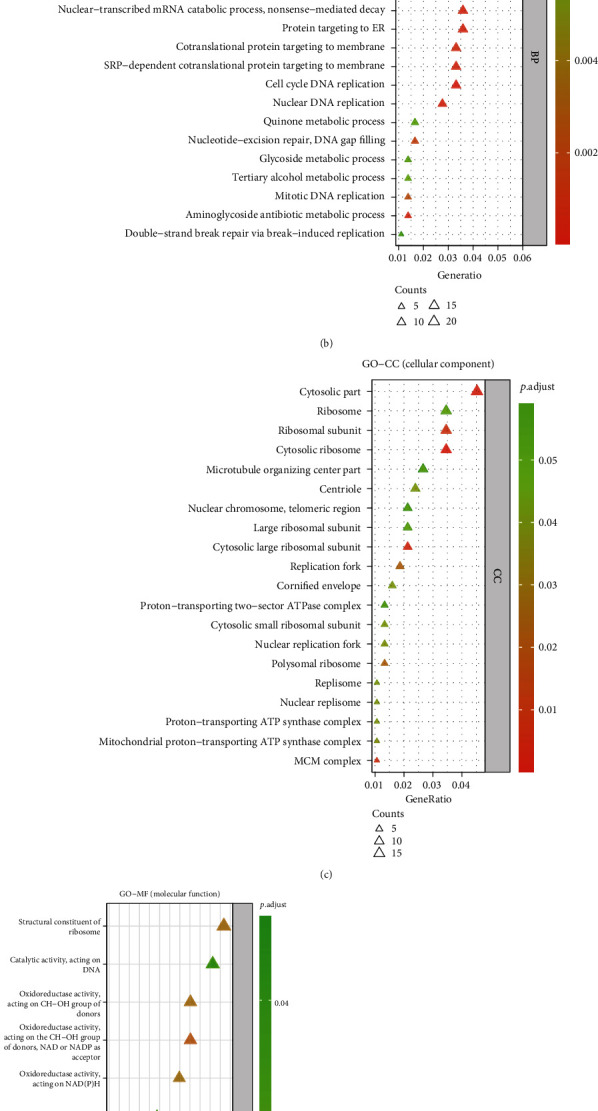
The biological functions enriched by TP73-correlated genes. (a) A heat map showed the top 10 positive and negative correlated genes in HNSC samples. (b) GO-BP analysis of significant TP73-related genes. (c) GO-CC analysis of significant TP73-related genes. (d) GO-MF analysis of significant TP73-related genes. (e) KEGG pathways analysis of significant TP73-related genes.

The top correlated genes ranked by the ascending and descending order of cor-Pearson values were obtained and their expression patterns in HNSC were shown in a heatmap. (Figure 9(a)). The top 10 positively correlated genes of TP73 were listed as follows: TXLNA (Taxilin Alpha), SAP30L (SAP30 Like), PRDM15 (PR/SET Domain 15), CDKN2C (Cyclin Dependent Kinase Inhibitor 2C), TSC2 (TSC Complex Subunit 2), SSBP3 (Single Stranded DNA Binding Protein 3), EDARADD (EDAR Associated Death Domain), TCEANC2 (Transcription Elongation Factor A N-Terminal And Central Domain Containing 2), CYB5RL (Cytochrome B5 Reductase Like), and FAM53B (Family With Sequence Similarity 53 Member B). The top 10 negatively correlated genes of TP73 were listed as follows: UPP1 (Uridine Phosphorylase 1), FOSL1 (FOS Like 1, AP-1 Transcription Factor Subunit), TM4SF19 (Transmembrane 4 L Six Family Member 19), PPIF (Peptidylprolyl Isomerase F), RPL22L1 (Ribosomal Protein L22 Like 1), PTS (6-Pyruvoyltetrahydropterin Synthase), KLK8 (Kallikrein Related Peptidase 8), ATP5MPL (ATP Synthase Membrane Subunit J), S100A10 (S100 Calcium Binding Protein A10), and NQO1 (NAD(P)H Quinone Dehydrogenase 1).

### 3.16. Bubble Charts to Identify the Biological Functions of the Significantly TP73-Correlated Genes

Bubble charts (Figure 9(b)) and Table 4 display the results concerning three GO term aspects (GO-BP, -CC, and -MF) and KEGG pathways. The significantly correlated genes of TP73 were primarily enriched in particular apoptotic-related BP, for example, DNA replication, G1/S transition of the mitotic cell cycle, and translational initiation (Figure 9(b)). The significantly correlated genes of TP73 were primarily enriched in the following ribosome-related CC: cytosolic part, ribosome, ribosomal subunit, and cytosolic ribosome (Figure 9(c)). The significantly correlated genes of TP73 were primarily enriched in certain MF, such as structural constituent of ribosome, catalytic activity (acting on DNA), oxidoreductase activity (acting on CH−OH group of donors), oxidoreductase activity (acting on the CH−OH group of donors, NAD or NADP as acceptor), and oxidoreductase activity (acting on NAD(P)H, Figure 9(d)). Five KEGG pathways were enriched, including DNA replication, ribosome, apoptosis, mismatch repair, and folate biosynthesis (Figure 9(e)). TP73 was reported to be related to some gene families, such as the Minichromosome Maintenance Complex family (MCM2, MCM3, MCM5, and MCM6), Ribosomal Protein Small (RPS12, RPS17, RPS25, and 29), and Large (RPL13, RPL24, RPL26, RPL27, RPL36, and RPL39) gene family (Figure 9(e)).

### 3.17. GSEA Analysis of TP73-Correlated Genes Functional Terms

**Figure 10:**
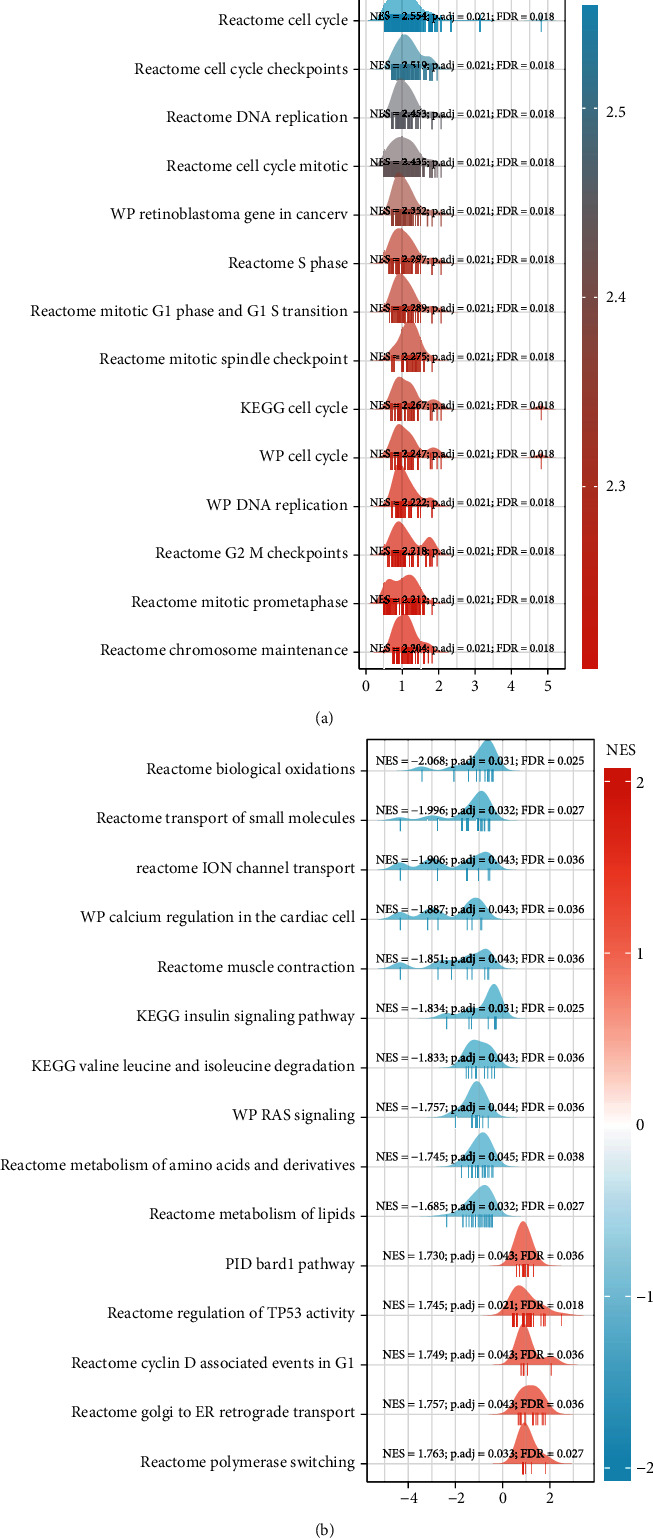
GSEA analysis to identify the functional terms enriched by the TP73-correlated genes. 30 functions, including all of the 10 functions with negative NES value, and top 20 functions with the highest positive NES value, were depicted by moutain plots.

According to mountain plots (Figure 10) and Table 2, cell cycle signaling was significantly enriched, including Mitotic G1 phase and G1s transition, G2 M checkpoint, and prometaphase and DNA replication pathways (Figure 10(a)). TP73 significantly negatively correlated genes were principally enriched in biological oxidation, insulin signaling pathway, ion channel transport, and RAS signaling pathways (Figure 10(b)).

### 3.18. Exploration of the Correlation between TP73 Expression and Immunity

**Figure 11:**
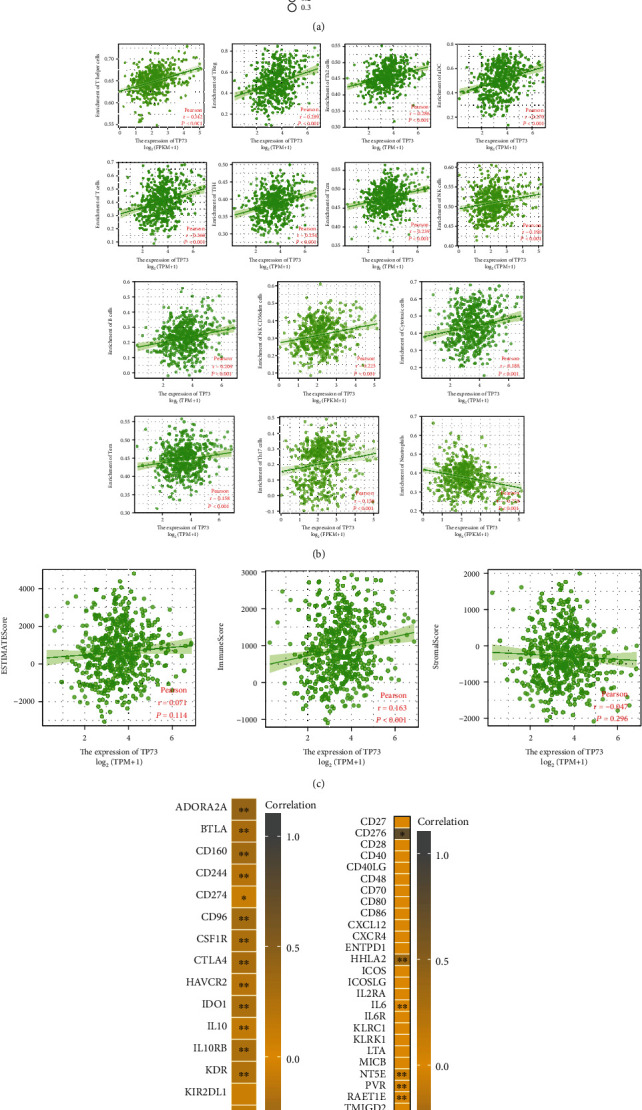
Exploration of the correlation between TP73 expression and immunity. (a) Lollipop plot visualized the correlation between TP73 expression and TIICs. (b) Association with the significant TP73-related TIICs was shown using scatter plots. (c) The correlation betweenTP73 gene and tumor microenvironment of HNSC by investigating the Estimate-Immune-Stromal score. (d) Association with the significant TP73-related immunosuppressive genes was shown using scatter plots. (e) Association with the significant TP73-related immunostimulatory genes was shown using scatter plots.

To further explore the relationship betweenTP73 gene expression and immunity, we analyzed TIICs, tumor microenvironment, and immune-related genes.  Figure 11(a) shows that TP73 had a substantial positive correlation with several TIICs, including T helper cells, Treg, Th2 cells, aDC, T cells, Th17 cells, TFH, Tcm, NK cells, B cells, NK CD56dim cells, Cytotoxic cells, and Tem. Additionally, there was a significant negative correlation between TP73 and neutrophils, a TIIC. For the TIICs strongly correlated with TP73 (*p* value <0.01), scatter plots were depicted (Figure 11(b)).

Table 5 showed the relationship between TP73 and marker sets of 16 TIICs in HNSC. Taken Tumor Associated Macrophage (TAM) as an example, the expression of TP73 was significantly positively correlated with a surface marker of TAM—chemokine factor CCL2 (*r* =0.148, *P* < 0.001), and another surface marker of TAM—cytokine IL10 (*r* =0.134, *P* = 0.003). The highest positive correlation was observed between TP73 expression level and a surface marker of Th17 cells—STAT3 (*r* =0.463, *P* < 0.001).

Figure 11(c) additionally explores the relationship between TP73 and the tumor immune microenvironment. The scatter plots found that stromal and estimate scores show no statistical significance (*P* > 0.05). However, TP73 was positively correlated with the tumor purity of HNSC according to the immune score (*r* =0.163, *P* < 0.001). In addition, 18 immunoinhibitor genes were positively correlated with TP73, including AORA2A, BTLA, CD160, CD244, CD98, CSF1R, CTLA4, HAVCR2, IDO1, IL10, IL10RB, KDR, KR2DL3, LAG3, LGALS9, PDCD1, TGFBR1, and TIGIT (Figure 11(d)). 6 immunostimulatory genes, CD276, HHLA2, IL6, NT5E, PVR, RAET1E, and TNFSF9, were negatively correlated with TP73 (Figure 11(e)).

## 4. Discussion

As shown in Figure 2(a), the frequency of TP73 genetic mutation was noted only in 1.3% of HNSC tumor samples, which was significantly lower than that for TP53 and TP63. In accordance with our findings, a previous research revealed the infrequent mutation of TP73 in HNSC with a C→A transversion mutation at codon 469 in C-terminal domain [25]. The combination of TP73 exon 2 G4C14-to-A4T14 and p53 intron three variant alleles was found to provide a significantly augmented risk of HNSC (OR =2.22, 95% CI: 1.08-4.56) in a sample drawn from the Italian population. Among participants less than 45 years old, individuals harboring TP73 exon 2 G4A variant allele had a 12.85-fold increased risk of HNSC when compared with people with the homozygous wild-type genotype (95% CI: 2.10-78.74) [26].

Our current research demonstrated that TP73 gene was overexpressed in HNSC tumor tissue as compared to control samples (Figures 3(g) and 3(h)). Previous immunohistochemistry results also identified an elevated expression of TP73 protein in HNSC tumor samples [27]. The current investigation demonstrated that the TP73 expression level was uncorrelated with the M stage representing metastatic status, or with lymphovascular invasion (Figure 5). In a contradictory finding, a HNSC patient cohort revealed a significant correlation between TP73 expression and distant metastasis and perineural/vascular invasion, suggesting an association between TP73 and an aggressive phenotype [27].

As a member of the TP53 family, TP73 has displayed different expression patterns and has diverse associations with prognosis in most human tumors [28, 29]. Previously, TP73 was proved to be an independent survival indicator in some cancers, such as lymphomas, leukemia, and gliomas. In the present study, increased TP73 indicated a better prognostic outcome for HNSC patients (Figure 6(c)). Similarly, downregulation of TP73 was associated with a worse OS in hematological malignancies of myeloid origin [10]. On the contrary, upregulated TP73 was found significantly associated with worse OS and DFS outcomes in laryngeal cancer [30]. Ribeiro showed that OSCC patients who had altered copy number of TP73 genes had a poor prognosis [31]. In gliomas, increased TP73 served as a stand-alone high-risk factor affecting the prognosis, while the downregulation of TP73 was closely related to favorable OS outcome [32].

PPI network analysis identified the top 10 hub genes which interacted with TP73, and eight of these were found significantly upregulated in HNSC. The expression levels of two genes, TP53 and MYC, showed no significant association with HNSC samples (Figure 8(b)). The interplay between TP73 and several associated genes (TP53, MYC, RB1, E2F1, and PPP1R13B) has been evidenced in previous literature. In TP53 mutant tumor cells, TAp73 could initiate the downstream pathway of TP53 and played a compensatory tumor suppressor role [33]. Serving as an oncogene, MYC may enlist TP73 to induce apoptosis in TP53-deficient tumor cells [34]. In addition, recent research indicated that TP73 inhibition was necessary for the anticancer effects of cyclin-dependent kinases RB1, thereby further leading to the cell cycle transition from G1 to S-phase [35]. Aberrations of cell cycle control were observed in HNSC, which resulted in downregulated activity of E2F transcription factors with concomitant enhanced cell cycle progression. TP73 activation via deregulated E2F1 activity may also serve as an anti-tumorigenic safeguard mechanism in HNSC independent of TP53 [36]. Another interacting gene shown in Figure 8(c), PPP1R13B, was found to enhance apoptotic function of TP63 and TP73 by selectively inducing the expression of endogenous TP53 target genes and further inhibiting tumor growth [37].

The results obtained by functional enrichment analysis and GESA analysis (Figures 9 and 10) showed that TP73 gene is mainly enriched in apoptosis-related pathways (e.g., DNA replication and cell cycle), insulin signaling pathway, Ras signaling, BARD1 pathway, regulation of p53 activity, biological oxidation, and ion channel transport. Functionally, TP73 protein can be divided into two subtypes, TAp73 and DNp73. The identical TAp73 isoforms produce markedly different effects on apoptosis and cell cycle arrest and were shown to act independently of TP53, thereby determining its transcriptionally activated apoptosis and growth arrest pathways. For example, TAp73-transfected osteosarcoma cells exhibited high levels of apoptosis induction at the mitochondrial level. In contrast, TAp73-transfected lung cancer cells exhibited high levels of late apoptosis, which was associated with high expression of death receptor pathway genes. In addition, TAp73 affected the cell cycle distribution of induced p21WAF1 mRNA in lung cancer cells but not in osteosarcoma cells [38]. A previous study regarding the involvement of TP73 in regulating reactive oxidative stress (ROS) showed that increased ROS observed in TAp73 knockout mice might result in the accumulation of mutations in proto-oncogenes, consequently contributing to increased genomic instability [39]. Deletion of the DNp73 played a key role in metabolic reprogramming and regression of p53-deficient malignancies, which was achieved by upregulating the IAPP gene. IAPP acted through the calcitonin receptor and RAMP3 to inhibit glycolysis, leading to ROS production as well as apoptosis. Another study proved that DNp73 synergizes with cMyc to promote the proliferation of primary mouse embryonic fibroblasts (MEFs) and inhibit P53-dependent apoptosis of MEFs. Moreover, an interaction between DNp73 and oncogenic ROS-induced fibrosarcomas in nude mice has been shown [40].

The current research also showed that TP73 was significantly positively correlated with TIICs, such as T cells, B cells, and NK cells (Figure 11(a)). Such findings are well supported by previous research. In nontumor lymphocytes, TP73 function has been found to be essential for antigen-induced circulating peripheral T cell death following the activation of T cell receptors in thymocytes [41]. Overexpression of TP73 is a common feature of B-cell chronic lymphocytic leukemia and may be involved in tumorigenesis by altering the ratio between oncogenic and anticancer gene variants of TP73 [42, 43]. NK cell malignancies exhibit specific promoter methylation patterns in which TP73 is consistently involved. These results suggest that TP73 may be an essential target in NK cell tumor transformation, and analysis of its methylation pattern may be a possible molecular tool for the detection of NK cell lymphoma [38]. To our knowledge, there is no report investigating the interplay between TP73 and tumor immune cells in the context of HNSC; thus, the present data provide a novel research direction for future studies.

It is noteworthy to highlight the limitation of the current research. Although we have designed a variety of bioinformatic analyses to illustrate the implication of TP73 in HNSC, we did not perform experimental validation of the findings predicted by this analysis. Future research can include cellular experiments using genetic transfection assays to observe the alteration of TP73 expression in HNSC cell lines. In addition, subsequent research could include the establishment of a subcutaneous tumor model in rats in order to examine the role of TP73 in the proliferation and migration of tumors in vivo. Another possible research direction is a clinical study to validate the correlation between TP73 expression and clinicopathological factors in HNSC. Taken together, our research provides a solid theoretical basis for the implications of TP73 in HNSC and indicates research directions for future studies.

## 5. Conclusion

The findings identified upregulated TP73 as a valid prognostic predictor for HNSC. Furthermore, the anti-tumor role of TP73 gene in HNSC was linked to mainly five pathways including DNA replication, ribosome, apoptosis, mismatch repair, and folate biosynthesis. This bioinformatics study highlights a possible biomarker role of TP73 and its therapeutic potential for treating HNSC.

## Figures and Tables

**Table 1:** Analysis of clinical characteristics of TCGA-HNSC patients according to TP73 gene.

Characteristic	Low expression of TP73	High expression of TP73	*P*
*n*	251	251	
T stage, *n* (%)			0.259
T1	14 (2.9%)	19 (3.9%)	
T2	63 (12.9%)	81 (16.6%)	
T3	70 (14.4%)	61 (12.5%)	
T4	94 (19.3%)	85 (17.5%)	
N stage, *n* (%)			0.502
N0	125 (26%)	114 (23.8%)	
N1	40 (8.3%)	40 (8.3%)	
N2	69 (14.4%)	85 (17.7%)	
N3	4 (0.8%)	3 (0.6%)	
M stage, *n* (%)			1.000
M0	233 (48.8%)	239 (50.1%)	
M1	2 (0.4%)	3 (0.6%)	
Clinical stage, *n* (%)			0.630
Stage I	9 (1.8%)	10 (2%)	
Stage II	48 (9.8%)	47 (9.6%)	
Stage III	56 (11.5%)	46 (9.4%)	
Stage IV	129 (26.4%)	143 (29.3%)	
Radiation therapy, *n* (%)			0.463
No	73 (16.6%)	81 (18.4%)	
Yes	148 (33.6%)	139 (31.5%)	
Primary therapy outcome, *n* (%)			0.104
PD	27 (6.5%)	14 (3.3%)	
SD	3 (0.7%)	3 (0.7%)	
PR	4 (1%)	2 (0.5%)	
CR	171 (40.9%)	194 (46.4%)	
Gender, *n* (%)			0.762
Female	65 (12.9%)	69 (13.7%)	
Male	186 (37.1%)	182 (36.3%)	
Race, *n* (%)			0.786
Asian	4 (0.8%)	6 (1.2%)	
Black or African American	22 (4.5%)	25 (5.2%)	
White	213 (43.9%)	215 (44.3%)	
Age, *n* (%)			0.067
≤60	112 (22.4%)	133 (26.5%)	
>60	139 (27.7%)	117 (23.4%)	
Histologic grade, *n* (%)			0.011
G1	40 (8.3%)	22 (4.6%)	
G2	154 (31.9%)	146 (30.2%)	
G3	50 (10.4%)	69 (14.3%)	
G4	0 (0%)	2 (0.4%)	
Smoker, *n* (%)			0.702
No	53 (10.8%)	58 (11.8%)	
Yes	192 (39%)	189 (38.4%)	
Alcohol history, *n* (%)			0.743
No	82 (16.7%)	76 (15.5%)	
Yes	166 (33.8%)	167 (34%)	
Lymphovascular invasion, *n* (%)			1.000
No	118 (34.6%)	101 (29.6%)	
Yes	65 (19.1%)	57 (16.7%)	
Lymphnode neck dissection, *n* (%)			<0.001
No	29 (5.8%)	61 (12.2%)	
Yes	221 (44.3%)	188 (37.7%)	
OS event, *n* (%)			0.059
Alive	131 (26.1%)	153 (30.5%)	
Dead	120 (23.9%)	98 (19.5%)	
DSS event, *n* (%)			0.030
Alive	165 (34.6%)	182 (38.2%)	
Dead	77 (16.1%)	53 (11.1%)	
PFI event, *n* (%)			0.013
Alive	140 (27.9%)	168 (33.5%)	
Dead	111 (22.1%)	83 (16.5%)	
Age, median (IQR)	62 (53, 69)	60 (53, 68)	0.198

**Table 2:** Analysis of TP73-correlated genes functional terms.

ID	Description	setSize	enrichmentScore	NES	*P* value	p.adjust	*q* values	Rank	leading_edge
REACTOME_CELL_CYCLE	REACTOME_CELL_CYCLE	234	0.655	2.554	0.001	0.021	0.018	592	Tags=53%, list=19%, signal=46%
REACTOME_CELL_CYCLE_MITOTIC	REACTOME_CELL_CYCLE_MITOTIC	183	0.645	2.435	0.001	0.021	0.018	592	Tags=55%, list=19%, signal=48%
REACTOME_M_PHASE	REACTOME_M_PHASE	115	0.599	2.161	0.001	0.021	0.018	592	Tags=50%, list=19%, signal=42%
REACTOME_TRANSCRIPTIONAL_REGULATION_BY_TP53	REACTOME_TRANSCRIPTIONAL_REGULATION_BY_TP53	114	0.491	1.766	0.001	0.021	0.018	698	Tags=34%, list=22%, signal=28%
REACTOME_DNA_REPAIR	REACTOME_DNA_REPAIR	111	0.603	2.159	0.001	0.021	0.018	857	Tags=59%, list=27%, signal=44%
REACTOME_CELL_CYCLE_CHECKPOINTS	REACTOME_CELL_CYCLE_CHECKPOINTS	89	0.723	2.519	0.001	0.021	0.018	439	Tags=57%, list=14%, signal=51%
REACTOME_MITOTIC_PROMETAPHASE	REACTOME_MITOTIC_PROMETAPHASE	82	0.640	2.212	0.001	0.021	0.018	592	Tags=56%, list=19%, signal=47%
REACTOME_SIGNALING_BY_RHO_GTPASES	REACTOME_SIGNALING_BY_RHO_GTPASES	94	0.512	1.790	0.001	0.021	0.018	288	Tags=33%, list=9%, signal=31%
REACTOME_REGULATION_OF_TP53_ACTIVITY	REACTOME_REGULATION_OF_TP53_ACTIVITY	68	0.519	1.745	0.002	0.021	0.018	698	Tags=37%, list=22%, signal=29%
REACTOME_DNA_DOUBLE_STRAND_BREAK_REPAIR	REACTOME_DNA_DOUBLE_STRAND_BREAK_REPAIR	66	0.642	2.150	0.002	0.021	0.018	818	Tags=62%, list=26%, signal=47%
REACTOME_RHO_GTPASE_EFFECTORS	REACTOME_RHO_GTPASE_EFFECTORS	65	0.587	1.960	0.002	0.021	0.018	526	Tags=51%, list=17%, signal=43%
REACTOME_MITOTIC_G1_PHASE_AND_G1_S_TRANSITION	REACTOME_MITOTIC_G1_PHASE_AND_G1_S_TRANSITION	51	0.714	2.289	0.002	0.021	0.018	474	Tags=63%, list=15%, signal=54%
REACTOME_MITOTIC_G2_G2_M_PHASES	REACTOME_MITOTIC_G2_G2_M_PHASES	51	0.564	1.809	0.002	0.021	0.018	592	Tags=57%, list=19%, signal=47%
WP_DNA_IRDAMAGE_AND_CELLULAR_RESPONSE_VIA_ATR	WP_DNA_IRDAMAGE_AND_CELLULAR_RESPONSE_VIA_ATR	49	0.651	2.082	0.002	0.021	0.018	687	Tags=57%, list=22%, signal=45%
REACTOME_RESOLUTION_OF_SISTER_CHROMATID_COHESION	REACTOME_RESOLUTION_OF_SISTER_CHROMATID_COHESION	46	0.696	2.196	0.002	0.021	0.018	463	Tags=57%, list=15%, signal=49%
REACTOME_BIOLOGICAL_OXIDATIONS	REACTOME_BIOLOGICAL_OXIDATIONS	20	-0.718	-2.068	0.003	0.031	0.025	547	Tags=60%, list=18%, signal=50%
REACTOME_TRANSPORT_OF_SMALL_MOLECULES	REACTOME_TRANSPORT_OF_SMALL_MOLECULES	54	-0.560	-1.996	0.003	0.032	0.027	416	Tags=31%, list=13%, signal=28%
REACTOME_ION_CHANNEL_TRANSPORT	REACTOME_ION_CHANNEL_TRANSPORT	17	-0.691	-1.906	0.005	0.043	0.036	153	Tags=41%, list=5%, signal=39%
WP_CALCIUM_REGULATION_IN_THE_CARDIAC_CELL	WP_CALCIUM_REGULATION_IN_THE_CARDIAC_CELL	17	-0.684	-1.887	0.005	0.043	0.036	206	Tags=41%, list=7%, signal=39%
REACTOME_MUSCLE_CONTRACTION	REACTOME_MUSCLE_CONTRACTION	14	-0.716	-1.851	0.005	0.043	0.036	379	Tags=57%, list=12%, signal=50%
KEGG_INSULIN_SIGNALING_PATHWAY	KEGG_INSULIN_SIGNALING_PATHWAY	21	-0.628	-1.834	0.003	0.031	0.025	778	Tags=48%, list=25%, signal=36%
KEGG_VALINE_LEUCINE_AND_ISOLEUCINE_DEGRADATION	KEGG_VALINE_LEUCINE_AND_ISOLEUCINE_DEGRADATION	11	-0.749	-1.833	0.005	0.043	0.036	489	Tags=82%, list=16%, signal=69%
WP_RAS_SIGNALING	WP_RAS_SIGNALING	28	-0.564	-1.757	0.005	0.044	0.036	356	Tags=29%, list=11%, signal=26%
REACTOME_METABOLISM_OF_AMINO_ACIDS_AND_DERIVATIVES	REACTOME_METABOLISM_OF_AMINO_ACIDS_AND_DERIVATIVES	36	-0.534	-1.745	0.005	0.045	0.038	381	Tags=42%, list=12%, signal=37%
REACTOME_METABOLISM_OF_LIPIDS	REACTOME_METABOLISM_OF_LIPIDS	107	-0.426	-1.685	0.003	0.032	0.027	526	Tags=27%, list=17%, signal=23%
PID_BARD1_PATHWAY	PID_BARD1_PATHWAY	19	0.669	1.730	0.005	0.043	0.036	504	Tags=53%, list=16%, signal=44%
REACTOME_REGULATION_OF_TP53_ACTIVITY	REACTOME_REGULATION_OF_TP53_ACTIVITY	68	0.519	1.745	0.002	0.021	0.018	698	Tags=37%, list=22%, signal=29%
REACTOME_CYCLIN_D_ASSOCIATED_EVENTS_IN_G1	REACTOME_CYCLIN_D_ASSOCIATED_EVENTS_IN_G1	10	0.783	1.749	0.005	0.043	0.036	370	Tags=60%, list=12%, signal=53%
REACTOME_GOLGI_TO_ER_RETROGRADE_TRANSPORT	REACTOME_GOLGI_TO_ER_RETROGRADE_TRANSPORT	29	0.618	1.757	0.005	0.043	0.036	463	Tags=48%, list=15%, signal=41%
REACTOME_POLYMERASE_SWITCHING	REACTOME_POLYMERASE_SWITCHING	10	0.789	1.763	0.003	0.033	0.027	338	Tags=70%, list=11%, signal=63%

**Table 3:** The correlation between TP73 expression and methylation sites in HNSC.

Sites	*r* value	*P* value
cg22614891[TSS-1918]	0.376	<0.001
cg18021902[TSS-1962]	0.311	<0.001
cg19135761[TSS-1867]	0.221	<0.001
cg26128092[TSS-1531]	0.171	<0.001
cg07174627[TSS-2312]	0.157	<0.001
cg11504517[TSS+818]	-0.234	<0.001
cg05924583[TSS+543]	-0.181	<0.001
cg16741710[TSS+305]	-0.176	<0.001
cg24073122[TSS-1095]	-0.145	0.001
cg21000072[TSS-1673]	0.127	0.004
cg20611911[TSS-1151]	-0.119	0.008
cg04021697[TSS-1778]	0.116	0.009
cg00565688[TSS-869]	-0.114	0.011
cg06782351[TSS-2366]	0.113	0.011
cg20677901[TSS-871]	-0.11	0.014
cg06262497[TSS-4405]	-0.109	0.015
cg17496659[TSS-836]	-0.108	0.016
cg13501117[TSS-2504]	0.104	0.02
cg04391111[TSS-1077]	-0.102	0.022
cg00295572[TSS-845]	-0.102	0.023
cg07382920[TSS-1435]	0.101	0.024
cg01915516[TSS-838]	-0.094	0.036
cg14781922[TSS-957]	-0.094	0.036
cg10143426[TSS-927]	-0.09	0.044
cg16823083[TSS-1669]	0.082	0.068
cg00780805[TSS-2380]	-0.078	0.082
cg17801268[TSS-2284]	0.061	0.176
cg09798435[TSS-2777]	0.057	0.206
cg18279839[TSS-2337]	-0.056	0.211
cg21012455[TSS-1972]	0.054	0.229
cg01434649[TSS-1211]	-0.052	0.243
cg07901143[TSS-374]	-0.05	0.261
cg25731359[TSS-1343]	0.05	0.264
cg01101400[TSS-2377]	-0.041	0.357
cg25885108[TSS-412]	-0.04	0.376
cg24336278[TSS-380]	-0.035	0.441
cg15120141[TSS-2596]	-0.031	0.492
cg12475507[TSS-1229]	-0.027	0.542
cg24678611[TSS-1349]	-0.027	0.545
cg06839380[TSS-2318]	-0.021	0.635
cg16406833[TSS-371]	-0.019	0.677
cg04344205[TSS-2263]	0.012	0.791
cg11729413[TSS-389]	-0.008	0.857
cg10038618[TSS-1362]	0.007	0.875
cg01530317[TSS-2502]	0.004	0.926
cg02153614[TSS-359]	0.004	0.936
cg04865841[TSS-2089]	Lack	Lack
cg11256802[TSS-2118]	Lack	Lack
cg22822803[TSS-2131]	Lack	Lack

**Table 4:** Biological functions of the significantly TP73-correlated genes.

Ontology	ID	Description	GeneRatio	BgRatio	*P* value	p.adjust	*q* value	geneID	Count
BP	GO:0044786	Cell cycle DNA replication	12/362	71/18670	1.078E-08	3.864E-05	3.693E-05	LIG1/MCM2/MCM3/MCM5/MCM6/POLA1/POLD1/POLE/RFC1/RPA2/ZPR1/E2F8	12
BP	GO:0033260	Nuclear DNA replication	10/362	60/18670	2.113E-07	0.0003699	0.0003535	LIG1/MCM2/MCM3/MCM5/MCM6/POLA1/POLD1/POLE/RFC1/RPA2	10
BP	GO:0006260	DNA replication	20/362	274/18670	4.358E-07	0.0003699	0.0003535	BARD1/LIG1/MCM2/MCM3/MCM5/MCM6/NFIA/POLA1/POLD1/POLE/RBBP4/RFC1/RPA2/S100A11/CHAF1B/ZPR1/E2F8/RMI1/RAD9B/GEN1	20
BP	GO:0045047	Protein targeting to ER	13/362	118/18670	4.907E-07	0.0003699	0.0003535	RPL13/RPL26/RPL27/RPL38/RPL39/RPL36A/RPS12/RPS17/RPS24/RPS25/RPS29/SEC61G/RPL36	13
BP	GO:0000184	Nuclear-transcribed mRNA catabolic process, nonsense-mediated decay	13/362	120/18670	5.967E-07	0.0003699	0.0003535	EXOSC10/RPL13/RPL26/RPL27/RPL38/RPL39/RPL36A/RPS12/RPS17/RPS24/RPS25/RPS29/RPL36	13
BP	GO:0030647	Aminoglycoside antibiotic metabolic process	5/362	10/18670	6.202E-07	0.0003699	0.0003535	AKR1C4/AKR1C1/AKR1C2/AKR1C3/AKR1B10	5
BP	GO:0072599	Establishment of protein localization to endoplasmic reticulum	13/362	122/18670	7.226E-07	0.0003699	0.0003535	RPL13/RPL26/RPL27/RPL38/RPL39/RPL36A/RPS12/RPS17/RPS24/RPS25/RPS29/SEC61G/RPL36	13
BP	GO:0006614	SRP-dependent cotranslational protein targeting to membrane	12/362	105/18670	9.104E-07	0.0004078	0.0003897	RPL13/RPL26/RPL27/RPL38/RPL39/RPL36A/RPS12/RPS17/RPS24/RPS25/RPS29/RPL36	12
BP	GO:0006613	Cotranslational protein targeting to membrane	12/362	109/18670	1.364E-06	0.0005429	0.0005189	RPL13/RPL26/RPL27/RPL38/RPL39/RPL36A/RPS12/RPS17/RPS24/RPS25/RPS29/RPL36	12
BP	GO:0006297	Nucleotide-excision repair, DNA gap filling	6/362	23/18670	3.893E-06	0.001395	0.0013332	LIG1/POLD1/POLE/RFC1/RPA2/XRCC1	6
BP	GO:0070972	Protein localization to endoplasmic reticulum	13/362	147/18670	5.926E-06	0.0019304	0.0018448	RPL13/RPL26/RPL27/RPL38/RPL39/RPL36A/RPS12/RPS17/RPS24/RPS25/RPS29/SEC61G/RPL36	13
BP	GO:1902969	Mitotic DNA replication	5/362	15/18670	6.822E-06	0.002037	0.0019467	LIG1/MCM2/MCM3/MCM6/POLA1	5
BP	GO:0006261	DNA-dependent DNA replication	13/362	153/18670	9.182E-06	0.0025306	0.0024184	LIG1/MCM2/MCM3/MCM5/MCM6/POLA1/POLD1/POLE/RFC1/RPA2/ZPR1/E2F8/GEN1	13
BP	GO:1902644	Tertiary alcohol metabolic process	5/362	19/18670	2.478E-05	0.0060658	0.005797	AKR1C4/AKR1C1/AKR1C2/AKR1C3/AKR1B10	5
BP	GO:0006413	Translational initiation	14/362	193/18670	2.539E-05	0.0060658	0.005797	EIF4EBP1/MTOR/RPL13/RPL26/RPL27/RPL38/RPL39/RPL36A/RPS12/RPS17/RPS24/RPS25/RPS29/RPL36	14
BP	GO:1901661	Quinone metabolic process	6/362	32/18670	3.016E-05	0.0067543	0.006455	AKR1C4/AKR1C1/AKR1C2/AKR1C3/AKR1B10/CYP4F11	6
BP	GO:0016137	Glycoside metabolic process	5/362	20/18670	3.252E-05	0.0067919	0.0064909	AKR1C4/AKR1C1/AKR1C2/AKR1C3/AKR1B10	5
BP	GO:0000082	G1/S transition of mitotic cell cycle	17/362	279/18670	3.412E-05	0.0067919	0.0064909	ANXA1/CASP2/CDKN2C/DHFR/EIF4EBP1/MCM2/MCM3/MCM5/MCM6/POLA1/POLE/RBL1/RPA2/RPL26/ZPR1/E2F8/DCUN1D3	17
BP	GO:0000727	Double-strand break repair via break-induced replication	4/362	11/18670	4.119E-05	0.0077523	0.0074088	MCM2/MCM3/MCM5/MCM6	4
BP	GO:0019083	Viral transcription	13/362	177/18670	4.327E-05	0.0077523	0.0074088	RPL13/RPL26/RPL27/RPL38/RPL39/RPL36A/RPS12/RPS17/RPS24/RPS25/RPS29/NUP62/RPL36	13
CC	GO:0022626	Cytosolic ribosome	13/376	112/19717	2.22E-07	8.74E-05	8.22E-05	RPL13/RPL26/RPL27/RPL38/RPL39/RPL36A/RPS12/RPS17/RPS24/RPS25/RPS29/RPL36/RPL22L1	13
CC	GO:0044445	Cytosolic part	17/376	247/19717	5.73E-06	0.00113	0.001062	EIF4EBP1/MTOR/RPL13/RPL26/RPL27/RPL38/RPL39/RPL36A/RPS12/RPS17/RPS24/RPS25/RPS29/TSC2/RPL36/CIAO2B/RPL22L1	17
CC	GO:0022625	Cytosolic large ribosomal subunit	8/376	63/19717	2.52E-05	0.003307	0.00311	RPL13/RPL26/RPL27/RPL38/RPL39/RPL36A/RPL36/RPL22L1	8
CC	GO:0042555	MCM complex	4/376	12/19717	5.71E-05	0.005622	0.005287	MCM2/MCM3/MCM5/MCM6	4
CC	GO:0044391	Ribosomal subunit	13/376	190/19717	7.63E-05	0.006011	0.005653	RPL13/RPL26/RPL27/RPL38/RPL39/RPL36A/RPS12/RPS17/RPS24/RPS25/RPS29/RPL36/RPL22L1	13
CC	GO:0042788	Polysomal ribosome	5/376	32/19717	0.000324	0.020848	0.019606	RPL38/RPL39/RPL36A/RPS29/RPL36	5
CC	GO:0005657	Replication fork	7/376	70/19717	0.00037	0.020848	0.019606	DNMT1/MCM3/POLA1/POLD1/RFC1/RPA2/ZMIZ2	7
CC	GO:0005753	Mitochondrial proton-transporting ATP synthase complex	4/376	22/19717	0.000725	0.035719	0.033591	ATP5F1E/ATP5MPL/PPIF/ATP5MD	4
CC	GO:0045259	Proton-transporting ATP synthase complex	4/376	23/19717	0.000865	0.037488	0.035254	ATP5F1E/ATP5MPL/PPIF/ATP5MD	4
CC	GO:0043601	Nuclear replisome	4/376	24/19717	0.001022	0.037488	0.035254	MCM3/POLA1/POLD1/RPA2	4
CC	GO:0043596	Nuclear replication fork	5/376	41/19717	0.001047	0.037488	0.035254	MCM3/POLA1/POLD1/RPA2/ZMIZ2	5
CC	GO:0030894	Replisome	4/376	26/19717	0.001396	0.038871	0.036555	MCM3/POLA1/POLD1/RPA2	4
CC	GO:0005814	Centriole	9/376	139/19717	0.001411	0.038871	0.036555	CCNF/PCNT/STIL/CEP135/CEP104/WRAP73/NEDD1/CEP120/KIF24	9
CC	GO:0022627	Cytosolic small ribosomal subunit	5/376	44/19717	0.001448	0.038871	0.036555	RPS12/RPS17/RPS24/RPS25/RPS29	5
CC	GO:0001533	Cornified envelope	6/376	65/19717	0.00148	0.038871	0.036555	ANXA1/PI3/SPRR1B/SPRR2G/SPRR4/LCE3E	6
CC	GO:0015934	Large ribosomal subunit	8/376	121/19717	0.002251	0.052932	0.049778	RPL13/RPL26/RPL27/RPL38/RPL39/RPL36A/RPL36/RPL22L1	8
CC	GO:0005840	Ribosome	13/376	272/19717	0.002284	0.052932	0.049778	RPL13/RPL26/RPL27/RPL38/RPL39/RPL36A/RPS12/RPS17/RPS24/RPS25/RPS29/RPL36/RPL22L1	13
CC	GO:0000784	Nuclear chromosome, telomeric region	8/376	125/19717	0.002757	0.05825	0.05478	MCM2/MCM3/MCM5/MCM6/POLD1/RPA2/XRCC1/CBX5	8
CC	GO:0016469	Proton-transporting two-sector ATPase complex	5/376	51/19717	0.002809	0.05825	0.05478	ATP5F1E/ATP6V1F/ATP5MPL/PPIF/ATP5MD	5
CC	GO:0044450	Microtubule organizing center part	10/376	185/19717	0.002993	0.058965	0.055452	CCNF/PCNT/STIL/CEP135/CEP104/WRAP73/SSX2IP/NEDD1/CEP120/KIF24	10
MF	GO:0004032	Alditol:NADP+1-oxidoreductase activity	6/361	13/17697	1.05E-07	3.66E-05	3.34E-05	AKR1C4/AKR1C1/AKR1C2/AKR1C3/AKR1B10/AKR1B15	6
MF	GO:0008106	Alcohol dehydrogenase (NADP+) activity	7/361	21/17697	1.26E-07	3.66E-05	3.34E-05	AKR1C4/AKR1C1/AKR1C2/AKR1C3/RDH8/AKR1B10/AKR1B15	7
MF	GO:0004033	Aldo-keto reductase (NADP) activity	7/361	26/17697	6.53E-07	0.000126	0.000115	AKR1C4/AKR1C1/AKR1C2/AKR1C3/RDH8/AKR1B10/AKR1B15	7
MF	GO:0016229	Steroid dehydrogenase activity	7/361	35/17697	5.7E-06	0.000827	0.000755	AKR1C4/AKR1C1/AKR1C2/HSD17B1/AKR1C3/RDH8/AKR1B15	7
MF	GO:0033764	Steroid dehydrogenase activity, acting on the CH-OH group of donors, NAD or NADP as acceptor	6/361	29/17697	2.21E-05	0.002561	0.002338	AKR1C4/AKR1C1/HSD17B1/AKR1C3/RDH8/AKR1B15	6
MF	GO:0052650	NADP-retinol dehydrogenase activity	4/361	11/17697	5.02E-05	0.00485	0.004428	AKR1C3/RDH8/AKR1B10/AKR1B15	4
MF	GO:0003688	DNA replication origin binding	5/361	24/17697	0.000106	0.00879	0.008024	MCM2/MCM3/MCM5/MCM6/POLA1	5
MF	GO:0016616	Oxidoreductase activity, acting on the CH-OH group of donors, NAD or NADP as acceptor	10/361	119/17697	0.000163	0.011845	0.010813	AKR1C4/AKR1C1/AKR1C2/HSD17B1/ME1/AKR1C3/RDH8/AKR1B10/PRXL2B/AKR1B15	10
MF	GO:0003735	Structural constituent of ribosome	13/361	202/17697	0.00027	0.017192	0.015694	RPL13/RPL26/RPL27/RPL38/RPL39/RPL36A/RPS12/RPS17/RPS24/RPS25/RPS29/RPL36/RPL22L1	13
MF	GO:0016614	Oxidoreductase activity, acting on CH-OH group of donors	10/361	128/17697	0.000296	0.017192	0.015694	AKR1C4/AKR1C1/AKR1C2/HSD17B1/ME1/AKR1C3/RDH8/AKR1B10/PRXL2B/AKR1B15	10
MF	GO:0016651	Oxidoreductase activity, acting on NAD(P)H	9/361	107/17697	0.000343	0.018063	0.01649	AKR1C4/AKR1C1/AKR1C2/NQO1/NDUFB2/TXN/AKR1C3/TXNDC17/CYB5RL	9
MF	GO:0008408	3′-5′ exonuclease activity	6/361	55/17697	0.000863	0.041702	0.038069	EXOSC10/POLD1/POLE/REXO2/EXO5/RAD9B	6
MF	GO:0016796	Exonuclease activity, active with either ribo- or deoxyribonucleic acids and producing 5′-phosphomonoesters	6/361	57/17697	0.001044	0.043249	0.039482	EXOSC10/POLD1/POLE/REXO2/EXO5/GEN1	6
MF	GO:0016895	Exodeoxyribonuclease activity, producing 5′-phosphomonoesters	4/361	23/17697	0.001109	0.043249	0.039482	POLD1/POLE/EXO5/GEN1	4
MF	GO:0016709	Oxidoreductase activity, acting on paired donors, with incorporation or reduction of molecular oxygen, NAD(P)H as one donor, and incorporation of one atom of oxygen	5/361	39/17697	0.001119	0.043249	0.039482	AKR1C4/AKR1C1/AKR1C2/AKR1C3/CYP4F11	5
MF	GO:0004303	Estradiol 17-beta-dehydrogenase activity	3/361	11/17697	0.00123	0.044091	0.04025	HSD17B1/RDH8/AKR1B15	3
MF	GO:0004527	Exonuclease activity	7/361	81/17697	0.001322	0.044091	0.04025	EXOSC10/POLD1/POLE/REXO2/EXO5/RAD9B/GEN1	7
MF	GO:0016655	Oxidoreductase activity, acting on NAD(P)H, quinone or similar compound as acceptor	6/361	60/17697	0.001368	0.044091	0.04025	AKR1C4/AKR1C1/AKR1C2/NQO1/NDUFB2/AKR1C3	6
MF	GO:0140097	Catalytic activity, acting on DNA	12/361	213/17697	0.001472	0.044502	0.040626	DNMT1/LIG1/MCM2/MCM3/MCM5/MCM6/POLA1/POLD1/POLE/XRCC1/EXO5/GEN1	12
MF	GO:0004529	Exodeoxyribonuclease activity	4/361	25/17697	0.001535	0.044502	0.040626	POLD1/POLE/EXO5/GEN1	4
KEGG	hsa03030	DNA replication	10/166	36/8076	1.65E-09	3.85E-07	3.6E-07	LIG1/MCM2/MCM3/MCM5/MCM6/POLA1/POLD1/POLE/RFC1/RPA2	10
KEGG	hsa03010	Ribosome	13/166	158/8076	2.04E-05	0.00239	0.002236	RPL13/RPL26/RPL27/RPL38/RPL39/RPL36A/RPS12/RPS17/RPS24/RPS25/RPS29/RPL36/RPL22L1	13
KEGG	hsa04210	Apoptosis	10/166	136/8076	0.000471	0.036711	0.03435	CASP2/CTSL/LMNB1/NGF/PIK3CB/TRAF2/TUBA4A/PIK3R3/MAP3K14/BCL2L11	10
KEGG	hsa03430	Mismatch repair	4/166	23/8076	0.001123	0.065715	0.061488	LIG1/POLD1/RFC1/RPA2	4
KEGG	hsa00790	Folate biosynthesis	4/166	26/8076	0.001808	0.084601	0.079159	DHFR/PTS/AKR1C3/AKR1B10	4

**Table 5:** The correlation between TP73 and surface markers of TIICs in HNSC.

Description	Gene markers	Correlation Pearson	*P* value
CD8+ T cell	CD8A	0.270	<0.001
	CD8B	0.239	<0.001
T cell (general)	CD3D	0.269	<0.001
	CD3E	0.334	<0.001
	CD2	0.329	<0.001
B cell	CD19	0.280	<0.001
	CD79A	0.262	<0.001
Monocyte	CD86	0.172	<0.001
	CSF1R	0.267	<0.001
TAM	CCL2	0.148	<0.001
	CD68	-0.064	0.153
	IL10	0.134	0.003
M1 macrophage	NOS2	0.326	<0.001
	IRF5	0.140	0.002
	PTGS2	0.050	0.266
M2 macrophage	CD163	0.056	0.209
	VSIG4	-0.010	0.823
	MS4A4A	0.062	0.163
Neutrophils	CEACAM8	0.154	<0.001
	ITGAM	0.320	<0.001
	CCR7	0.261	<0.001
Natural killer cell	KIR2DL1	0.081	0.071
	KIR2DL3	0.131	0.003
	KIR2DL4	0.217	<0.001
	KIR3DL1	0.115	0.010
	KIR3DL2	0.207	<0.001
	KIR3DL3	0.135	0.002
	KIR2DS4	0.067	0.132
Dendritic cell	HLA-DPB1	0.263	<0.001
	HLA-DQB1	0.262	<0.001
	HLA-DRA	0.303	<0.001
	HLA-DPA1	0.299	<0.001
	CD1C	0.233	<0.001
	NRP1	0.084	0.061
	ITGAX	0.095	0.034
Th1	TBX21	0.249	<0.001
	STAT4	0.179	<0.001
	STAT1	0.269	<0.001
	IFNG	0.224	<0.001
	TNF	0.178	<0.001
Th2	GATA3	0.224	<0.001
	STAT6	0.320	<0.001
	STAT5A	0.393	<0.001
	IL13	0.137	0.002
Tfh	BCL6	0.257	<0.001
	IL21	0.264	<0.001
Th17	STAT3	0.463	<0.001
	IL17A	0.294	<0.001
Treg	FOXP3	0.387	<0.001
	CCR8	0.330	<0.001
	STAT5B	0.188	<0.001
	TGFB1	-0.015	0.738
T cell exhaustion	PDCD1	0.296	<0.001
	CTLA4	0.280	<0.001
	LAG3	0.267	<0.001
	HAVCR2	0.204	<0.001
	GZMB	0.202	<0.001

## Data Availability

The data that support the findings of this study are available from the corresponding author upon reasonable request.
